# Milk from a Public Health Standpoint—City of Bristol
^1^This and the following papers on Milk were read at a meeting of the Society on February 10th.


**Published:** 1909-03

**Authors:** D. S. Davies

**Affiliations:** Medical Officer of Health for the City and County of Bristol


### MILK FROM A PUBLIC HEALTH STANDPOINT—CITY OF BRISTOL.[1]

BY

D. S. DAVIES, M.D. Lond., D.P.H.,

*Medical Officer of Health for the City and County of Bristol.*

---

THE city numbers 377,642 persons, and covers 17,289 acres or 27 square miles. The milk supply of the city is derived from 381 farms, through 1,000 dealers. Of the 381 farms, 81 are within the present city limits, and 300 outside the limits, scattered over the counties of Gloucester (134), Somerset (146) and Wiltshire (20). One inspector of dairies, cowsheds and milkshops is appointed to cover the whole of the city area, with a workman assistant, who revisits and reports neglected work, &c. In 1897 and 1904 the city extensions added a considerable rural area containing 79 dairy farms; 36 of these were added in 1904 to the existing city area, but the additional inspectors then advised for these districts have not yet been appointed. The Report of 1904 advising this is still under the Committee's consideration.

*By-laws and Regulations in force in the city.*

(*a*) The Dairies, Cowsheds and Milkshops Order, 1885, and the Amending Order of 1886, control the conditions of the milk service generally.

1 This and the following papers on Milk were read at a meeting of the Society on February 10th.

(*b*) Regulations made by the Council on October 23rd, 1905, prescribe details as to air space, lighting, ventilation, water supply, drainage, &c.

(*c*) The Bristol Corporation Act, 1905, deals specially with tuberculosis.

*Inspection, &c., in the city.*

The number of general inspections made in the city during 1907 was 2,379, and a similar number is paid in each year.

In addition special inspections by an Assistant Medical Officer have been ordered of all the dairy farms upon three occasions, viz. in 1897 and 1900, and the third special inspection, which includes also dairies and milkshops, is now in course of completion.

Considerable improvement has followed each of these special visits, especially at dairy farms which have from time to time been included in the city limits owing to extension of the boundaries.

In 1899 a systematic examination of the milk from all farms supplying the city was carried out by Professor Délépine. Out of seventy-four samples examined biologically only four showed the presence of tuberculosis. The samples were taken from the milk as brought into the city for delivery.

Milk contracts for the city and port hospitals contain clauses protective against tuberculosis.

The Health Committee has recently ordered a further special examination of the milk supplied to the city, which has been entrusted to University College Laboratory.

*Observations in regard to inspection of milk generally.*

Common sense should govern this as other matters upon which public sentiment is apt to run wild.

Cleanliness at dairy farms is essential: the most difficult point is to secure cleanliness of the cows' teats, second to this to secure cleanliness of the milkers' hands, and the special danger of typhoid fever from "carrier" milkers by hand infection is obvious.

Milk-carried outbreaks will not be entirely suppressed by complete methods of ordinary cleanliness, as they are often due to infection from very slight cases which are "missed," and so gain added opportunity for mischief.

Milk should be obtained, carried and retailed with as much attention to cleanliness as possible, but this will not remove the inherent inadvisability of consuming an animal secretion raw. All milk should be cooked, just as animal flesh is cooked.

*Milk and infant mortality.*

Absolute cleanliness, avoidance of preservative drugs, and care in handling and storing should mean some lessening in infant mortality; but domestic dealing with milk has to be reckoned with as well as the dealings of cow-keeper and dairy-man.

Also the cleanest of milk improperly given to infants as a substitute for mothers' milk, either in too concentrated a form or mixed with unsuitable food, will account for a proportion of illness.

Home advice with personal teaching is the most hopeful method to deal with this.

Relatively the infant mortality and the diarrhœal mortality in Bristol is satisfactory in comparison with the large towns.

TOWNS OVER 200,000.

1907.

| Name of Town. | Infant Mortality per 1,000 births. | Death Rate per 100,000 living from Diarrhœa. |
|---|---|---|
| London | 116 | 40 |
| Liverpool | 144 | 73 |
| Manchester | 146 | 50 |
| Birmingham | 147 | 43 |
| Leeds | 130 | 38 |
| Sheffield | 145 | 99 |
| **Bristol** | **100** | **32** |
| West Ham | 131 | 66 |
| Bradford | 124 | 17 |
| Newcastle-on-Tyne | 123 | 14 |
| Hull | 127 | 37 |
| Nottingham | 165 | 63 |
| Leicester | 131 | 31 |
| Salford | 140 | 44 |
| Portsmouth | 123 | 29 |

